# Ventilator-associated tracheobronchitis: pre-emptive, appropriate antibiotic therapy recommended

**DOI:** 10.1186/s13054-014-0627-5

**Published:** 2014-11-19

**Authors:** Donald E Craven, Jana Hudcova, Yuxiu Lei

**Affiliations:** Center for Infectious Diseases & Prevention, Lahey Medical Center & Hospital, 41 Mall Road, Burlington, MA 01805 USA; Tufts University School of Medicine, 800 Washington Street, Boston, MA 02115 USA; Department of Surgical Critical Care, Lahey Medical Center & Hospital, 41 Mall Road, Burlington, MA 01805 USA; Department of Pulmonary & Critical Care Medicine, Lahey Medical Center & Hospital, 41 Mall Road, Burlington, MA 01805 USA

## Abstract

Nseir and colleagues presented data from a large multicenter study of patients with ventilator-associated tracheobronchitis (VAT), demonstrating that appropriate antibiotic therapy for VAT was an independent predictor for reducing transition to pneumonia (ventilator-associated pneumonia, or VAP). These data added to the growing evidence supporting the use of appropriate antibiotic therapy for VAT as a standard of care to prevent VAP and improve patient outcomes.

## Introduction

In a previous issue of *Critical Care*, Nseir and colleagues [1] presented interesting data demonstrating that appropriate antibiotic therapy for ventilator-associated tracheobronchitis (VAT) reduced transition to ventilator-associated pneumonia (VAP). Of the 122 study patients, 8.1% developed VAT, of whom 13.9% later developed VAP. *Pseudomonas aeruginosa*, *Staphylococcus aureus*, and *Acinetobacter baumannii* were the most common pathogens isolated. In the multivariable analysis, appropriate antibiotic therapy was the only risk factor independently associated with reduced risk of transition to VAP (*P* =0.009).

Previous studies have demonstrated that patients diagnosed with VAT or VAP (or both) have increased ventilator days, length of ICU stay, and associated health-care costs [2-6]. Patients with VAT or VAP present with elevated temperature, leukocytosis, purulent sputum with many polymorphonuclear leukocytes on Gram stain, plus an endotracheal aspirate (ETA) culture having >10 moderate (+++) semi-quantitative growth, or a quantitative culture having at least 100,000 (at least 10^5^) organisms per milliliter of a bacterial pathogen [5,7]. However, VAP also requires a new and persistent infiltrate on chest x-ray. The fact that 10% to 30% of patients with VAT progress to VAP makes VAT an ideal target for antibiotic therapy to prevent VAP and improve patient outcomes [5,7,8]. Intravenous or aerosolized antibiotic therapy (or both) for VAT has been shown to reduce VAP, ventilator days, and length of ICU stay [3,4,9,10].

Bouza and colleagues [8] compared use of pre-emptive antibiotic therapy versus controls to prevent VAT and VAP in high-risk patients following major heart surgery. Forty patients were randomly assigned to a 3-day course of linezolid and meropenem versus 38 control patients followed for development of VAT, VAP or both. The antibiotic-treated group had significantly lower rates of VAT/VAP, 32/1,000 days versus controls 65/1,000 days (*P* <0.03), and a longer time to the first episode of VAT/VAP (9 versus 4.5 days, *P* =0.02).

Recently, there has been increased interest in the use of adjunctive aerosolized antibiotic therapy for VAT and VAP, administered by an improved nebulizer delivery system to increase weaning from the ventilator and shorten ICU stay [4,9,10]. A double-blind, placebo-controlled study is in progress to evaluate adjunctive aerosolized amikacin and fosfomycin therapy for the treatment of VAP due to Gram-negative bacilli and to decrease ventilator days and ICU stay [11].

**Figure 1:**
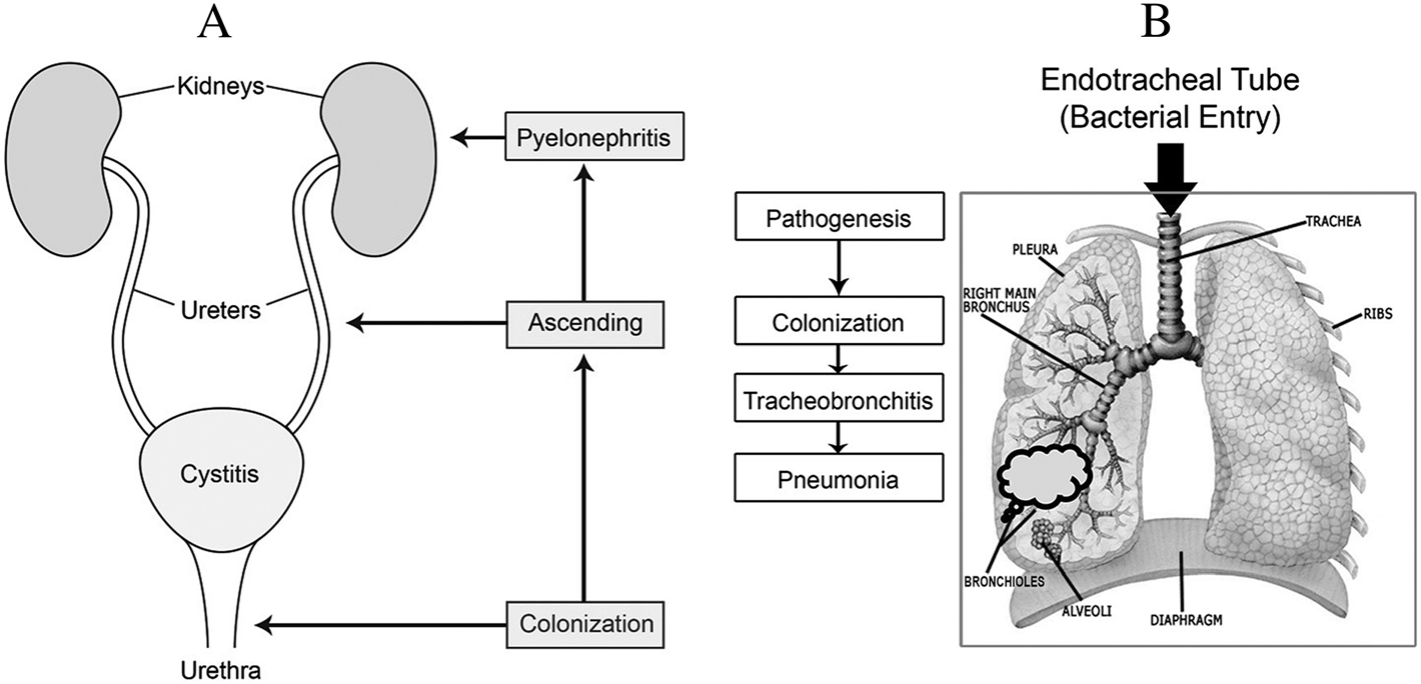
**Pathogenesis and routine antibiotic therapy for urinary tract infections versus ventilator-associated pneumonia and tracheobronchitis. (A)** The pathogenesis of ‘ascending’ urinary tract infection due to cystitis or pyelonephritis or both, which are currently treated with antibiotics as a ‘standard of care’. **(B)** The pathogenesis of ‘descending’, primarily ‘one-way’ respiratory tract infection manifests as ventilator-associated tracheobronchitis or pneumonia, for which we would recommend treatment with intravenous or aerosolized antibiotic therapy or both. Reprinted with permission from Lippincott Williams & Wilkins [12].

Interestingly, different approaches exist for antibiotic treatment of urinary tract infections (UTI) due to cystitis or pyelonephritis versus respiratory tract infections due to VAT and VAP (Figure 1A). Patients with UTI presenting with fever, leukocytosis, dysuria, and a urine culture having at least 100,000 bacterial pathogens per milliliter due to cystitis or pyelonephritis are treated with antibiotics as a standard of care and infected urine can be removed by voiding or bladder catheter. By comparison, VAT and VAP are ‘descending’ infections in a mechanically ventilated, intubated patient requiring removal of infected secretions by intermittent suctioning (Figure 1B). Leakage of oropharyngeal secretions around the endotracheal tube (ETT) or embolization of bacterial biofilm from the inner surface of the ETT to lower tracheobronchial and alveoli after airway instrumentation have been implicated in the pathogenesis of VAT to VAP. Also, bacterial colonization in ventilator tubing condensate can be inadvertently washed into the lung, especially with supine positioning. Intubated patients have decreased bacterial clearance due to reduced cough, sedation, and the presence of the ETT, all of which may increase bacterial lung burden and challenge lung cellular and humoral defenses. These significant clinical differences in pathogenesis between lung and urinary tract infections, in our opinion, add support for the use of pre-emptive appropriate intravenous or aerosolized antibiotic treatment (or both) for VAT or VAP as a standard of care [1,8,9].

Ventilated ICU patients developing VAT or VAP or both are at increased risk for chronic co-morbidities that include debility, tracheostomy, acute and chronic lung damage, delirium, post-traumatic stress disorder, and short- or long-term cognitive impairment that increase the need for rehabilitation, chronic care, hospital readmission, and increased health-care costs [13,14]. Unroe and colleagues [15] studied 99 ventilated patient survivors 1 year after hospital discharge and found increased need for chronic care, hospital readmission, multiple transitions of care, and increased health-care costs. At 1 year, only 9% of study patients were living independently, there were 150 hospital readmissions and numerous transitions to and from chronic care facilities, and health-care costs were estimated at $3.5 million per survivor.

In a recent survey of VAT involving medical staff in 288 ICUs in 16 different countries worldwide, 50.3% of respondents recommended antibiotic therapy for VAT, 93% thought VAT increased length of ICU stay, and 50% believed that VAT increased patient mortality [16]. Data from several randomized clinical trials and a meta-analysis support the use of pre-emptive appropriate antibiotic therapy for VAT to reduce progression to VAP, ventilator days, length of ICU stay, and associated health-care costs [3,4,6,8]. Assessing serial semi-quantitative ETA or quantitative ETA cultures allows identification of likely bacterial pathogens and antibiotic sensitivity data needed to initiate appropriate ‘targeted’ intravenous or aerosolized antibiotic therapy (or both), especially for infections due to *S. aureus*, *P. aeruginosa*, *Acinetobacter species*, or other multi-drug-resistant Gram-negative pathogens [17]. Considering the available data, we recommend that pre-emptive appropriate antibiotic therapy for VAT be considered a new standard of care.

## Abbreviations

ETA: Endotracheal aspirate
ETT: Endotracheal tube
UTI: Urinary tract infection
VAP: Ventilator-associated pneumonia
VAT: Ventilator-associated tracheobronchitis
